# Rebar Corrosion Investigation in Rubber Aggregate Concrete via the Chloride Electro-Accelerated Test

**DOI:** 10.3390/ma12060862

**Published:** 2019-03-14

**Authors:** Jian Liang, Han Zhu, Lei Chen, Xiaobin Han, Qinglin Guo, Ying Gao, Chunsheng Liu

**Affiliations:** 1 School of Civil Engineering, Tianjin University, Tianjin 300072, China; sdaulj@163.com (J.L.); hanzhu2000@tju.edu.cn (H.Z.); leichen2009@163.com (L.C.); gaoyhebeu@163.com (Y.G.); chunshl12@163.com (C.L.); 2 Anhui & Huaihe River Institute of Hydraulic Research, Hefei 230088, China; 3 School of Civil Engineering, Hebei University of Engineering, Handan 056038, China; guoql@hebeu.edu.cn

**Keywords:** rubber aggregate concrete, chloride ion diffusion, rebar corrosion, electro-accelerated corrosion test

## Abstract

Erosion effect of chloride ions from the marine, deicing salt, saline-alkali land and some industrial environments will cause the corrosion of rebar in concrete, which is one of the most harmful factors affecting the durability of concrete structure. It had been proved that the incorporation of rubber aggregates increase the capillary saturation of cement concrete and reduce the corrosion degree of rebar. This paper investigated the rebar corrosion behavior in rubber aggregate concrete under the chloride electro-accelerated corrosion condition and such an investigation has not been seen in any public literature. Two water-cement ratios (0.45 and 0.55) and four rubber contents (0, 50, 100, and 150 kg/m^3^) were selected for experiment. Four-point bending tests of concrete beam were conducted before and after chloride ion erosion in order to determine effect of rubber aggregate on the durability of rebar. Results showed that rebar corrosion degree decreased with the increase of rubber aggregate in concrete. Besides, the more the dosage of rubber is, the better the anti-crack performance of cement concrete. This will postpone the crack development and reduce the peak of rebar corrosion.

## 1. Introduction

Different types of concrete have been widely used in construction and water conservancy projects due to their different strength, different shapes, and good overall performance. However, rebar corrosion, the most commonly disease, caused by chloride ion erosion from the marine environment, deicing salt, saline-alkali land, and some industrial sites is a major threat to the durability of concrete structure. This also results huge direct and indirect economic losses. Research had showed that the corrosion rate of reinforced concrete was affected by the saturated degree of capillary pores. The use of rubber aggregates can improve the capillary pore saturation and reduce the rebar corrosion in the concrete. The weight loss rate decreased with the increase of rubber content.

For a long time, researchers have devoted themselves to improve the concrete strength. Unfortunately, shortcomings such as brittleness and cracks of concrete would rise prominently with the improvement of strength. In recent decades, scholars have begun to apply rubber particles in the concrete. This not only improves the deadweight, brittleness, and other disadvantages of concrete, but also expands the use of waste rubber as recycled resources. The rubber cement concrete is a composite material, which is made by mixing the waste rubber particles and base cement concrete. As a superelastic material, rubber improves the internal structure of concrete through physical action without changing the chemical properties of concrete. The rubber elastic modulus is almost negligible when compared with concrete. The rubber particles, uniformly distributes in the concrete and replaces part of the aggregate, can be seen as elastic holes to prevent the generation and development of micro cracks in the concrete, and forms the deformed center that absorbs strain energy. Many scholars have studied the physical and mechanical properties of rubber aggregate concrete and found that rubber particles can significantly improve the crack resistance of concrete. At the same time, rubber cement concrete will not suddenly undergo brittle failure when they are destroyed. In addition, rubber particles also have positive effects in improving the frost resistance of concrete and the migration of chloride ions. Pelisser et al. [1] studied that the density of rubber-mixed concrete was reduced by 13% compared with ordinary concrete. Yilmaz et al. [2] stated that the flexural strength increased 20% after the rubber was added into the concrete. However, the flexural strength showed a downward trend when rubber dosage was continued to increase. The control concrete exhibited a brittle fracture, but the rubber cement concrete was ductile fracture. Kang et al. [3] suggested that concrete containing rubber particles generated obvious plastic deformation during the bending, and did not undergo plastic fracture when it was subjected to maximum load, but rather suffered ductile failure after a large plastic deformation. Studies by Thomas et al. [4] showed that the concrete contains waste rubber had a smaller carbonization depth than the control concrete. Raghavan et al. [5] insisted that the 0.6% rubber concrete had the lowest mass loss after the freeze-thaw cycles. Oikonomou et al. [6] showed that the chloride ion diffusion decreased with increasing of rubber content. Gupta et al. [7] studied the mixing ratio of the rubber concrete on chloride ion diffusion. They found the chloride ion concentration in rubber concrete is very low, so the rubber aggregate concrete had a good chloride ion diffusion resistance. Thomas et al. [4] showed that the diffused depth of chloride ion in concrete which contained 10% rubber content was smaller than that of control concrete.

Thomas et al. [8] pointed out that the disposal of waste tire rubber had become an outstanding environmental issue in the world. The raw materials used in rubber cement concrete mainly from waste tires. This is a very effective method for protecting the land and reusing the waste rubber. At the same time, the development of rubber cement concrete has many economic and social benefits due to its low cost. On the other hand, China’s annual production of concrete in recent years is about 1.3 billion cubic meters. With the further increase of infrastructure construction in China, it can be foreseen that concrete production will be very large in the future. Both carbonization and chloride ion corrosion will reduce the durability of reinforced concrete. It is extremely serious that a large amount of concrete facing endurable and damaging consequences. Therefore, we need to find new solutions from the perspective of materials science. Rubber cement concrete has been paid extensive attention due to its excellent performance in terms of wear resistance [9], anti-carbonization [4], shrinkage, freeze-thaw [5], acid resistance and resistance to chloride ions [7].

Oikonomou et al. [6] found that with the increase of rubber in the mortar, the chloride ion diffusion also decreased, compared with the control group, the chloride ion diffusion was reduced by 14.22% and 35.85% for the rubber content 2.5% and 15%, respectively. Bravo et al. [10] conducted a chloride ion migration test, a higher chlorine diffusion coefficient was obtained when increased the rubber particles size. Onuaguluchi et al. [11] stated that the charge transfer ability was reduced by 5–10% when concrete was mixed with small pieces of rubber. Al-Akhras et al. [12] studied the properties of mortars containing rubber ash. During the curing process of 90 days, the charge of concrete was controlled to be 1875C, and the charge passed 5% and 10% rubber ash mortar was 520C and 35C, respectively. Gesoğlu et al. [13] have shown that the ions diffusion rate rised with the increase of rubber particles in the self-shrinking rubber concrete. Thomas et al. [4] showed that the diffusion depth and content of chloride ions in rubber concrete was small. Because the rubber particles are impermeable to moisture, and do not allow chloride ions to pass through.

It could be seen that the rubber aggregate was helpful to weaken the chloride ions diffusion, but effects of rubber content on the strength of concrete and the chloride ions diffused characteristic were unknown. Therefore, this paper investigated the corrosion mechanism of rebar in rubber cement concrete in the environment of chloride salt. Accelerated corrosion test was conducted using concrete beams with 0.45 and 0.55 water-cement ratios and different rubber contents. Electrochemically accelerated methods were used to simulate corrosion of the rebar in the etching medium (3.5% NaCl solution). The concrete weight loss rate and crack width after two corrosion cycles (5d, 10d) were evaluated. Finally, the flexural performance of concrete beams after corroding was tested. Bearing capacity, deflection and failure modes with different rubber contents before and after corroding were analyzed.

## 2. Experimental Design

**Figure 1:**
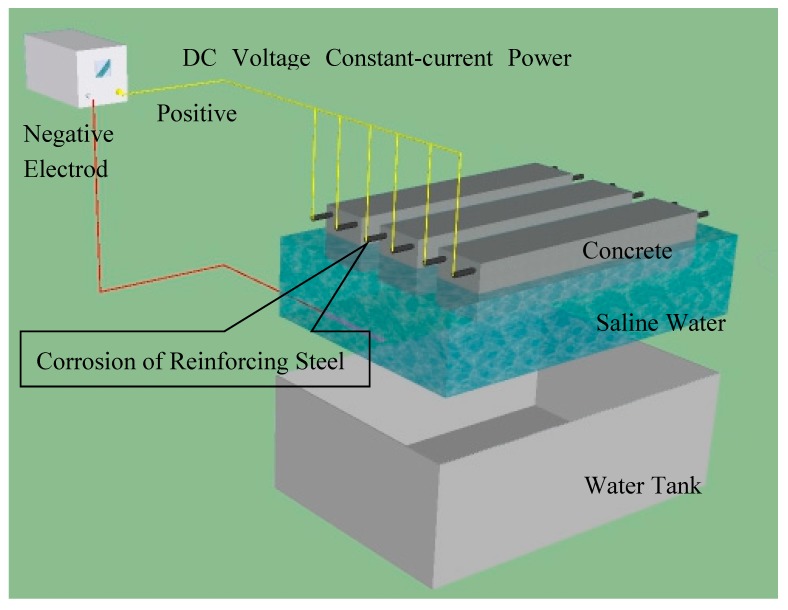
Accelerated corrosion test device.

The key of experiment is to form a corrosion current loop. Besides, it must be ensured that oxygen, water and chloride ions are present around the rebar. Almusallam et al. [14] used electro corrosion method to study the influence of rebar corrosion on the mechanical properties of concrete. Lee et al. [15] studied the relationship between rebar corrosion and its mechanical properties using electro corrosion quantitatively. Zhang et al. [16] used electrochemical methods to corrode rebar in reinforced concrete slab for tensile and fatigue behavior investigation. Wu et al. [17] studied the effect of two accelerated corrosion methods on the mechanical properties of corroded rebars by conducting tensile tests under artificial climate and galvanostatic accelerated conditions. Combined with the previous literatures on the accelerated corrosion of reinforced concrete [15,16,17,18,19], an accelerated test device was designed, as shown in Figure 1.

In the test, the voltage output of DC power is 0–30 V, the maximum output electric current is 5 A. Concrete specimens were beams with a scale 100 mm × 100 mm × 515 mm. Half of the beams in vertical direction were submerging in the 3.5% NaCl solution, Two rebar with a diameter of 5 mm were built in the part above water level. The concrete cover depth was 10 mm. As the anode, the rebar are connected to the positive pole of the power. The cathode is a stainless wire-steel arranged at the bottom of specimens. A resistance is connected in the loop, its value is 10.8 Ω. Concrete proportions are shown in Table 1. It should be stated that a high W/C ratio 0.55 was selected for experiment in order to ensure the good working performance of concrete.

All of the specimens were cured by 28 days firstly. After that they were taken out for accelerated corrosion. The corrosion periods 5 and 10 days was selected in this study. During the test, every rebar was connected with a resistance and then paralleled connected with power (Figure 1). A constant voltage 12 V was employed, and the current through the rebar changes with the resistance in the closed circuit. The voltage across the series resistor was measured by 6 h in order to calculate the current through the rebar in the beam. According to Faraday’s law, rebar corrosion depends on the quantity of electricity which had passed through the rebar, then the rebar corrosion can be estimated.

In this paper, evaluation on rebar corrosion was conducted according to standard GBT50344-2004. Half-cell potential method for electrochemical analysis was adopted. The corrosion instrument, DJXS-05 was adopted for potential test. Before measuring the potential, the applied electric field was turned off, and after measuring the potential, the applied electric field was restored. To avoid the variability of the potential, potential was measured until the power was switching off for 10 min. Meanwhile, repeated measurements of the potential were adopted to reduce the variability of the potential in the experiment. In accordance with standard GBT50344-2004, the measured potential was accepted if the error of two consecutive measurements was less than 10 mV. Otherwise, repeated measurements would be carried out until the error was less than the requirements of the specifications.

**Figure 2:**
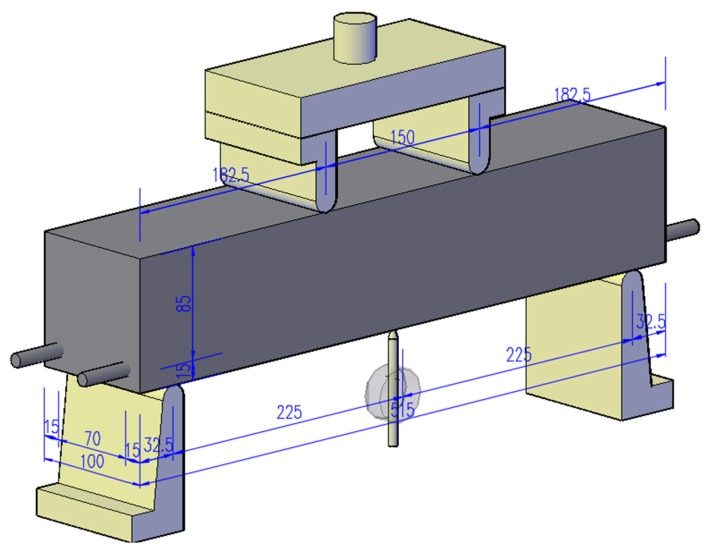
Loading test device (Unit: mm).

On that basis, a four-point bending test was conducted on the reinforced concrete beams, as shown in Figure 2. Load-deflection curves, deflections at mid-span and ultimate failure cracks distribution were selected estimate the corrosion on the mechanical properties of rubber aggregate concrete and control one. The test was carried out using a WE-30 universal material test machine at maximum load 100 KN and a loading speed of 0.3 cm/s.

## 3. Test Results and Discussion

### 3.1. Comparison on Specimen Corrosion

**Figure 3:**
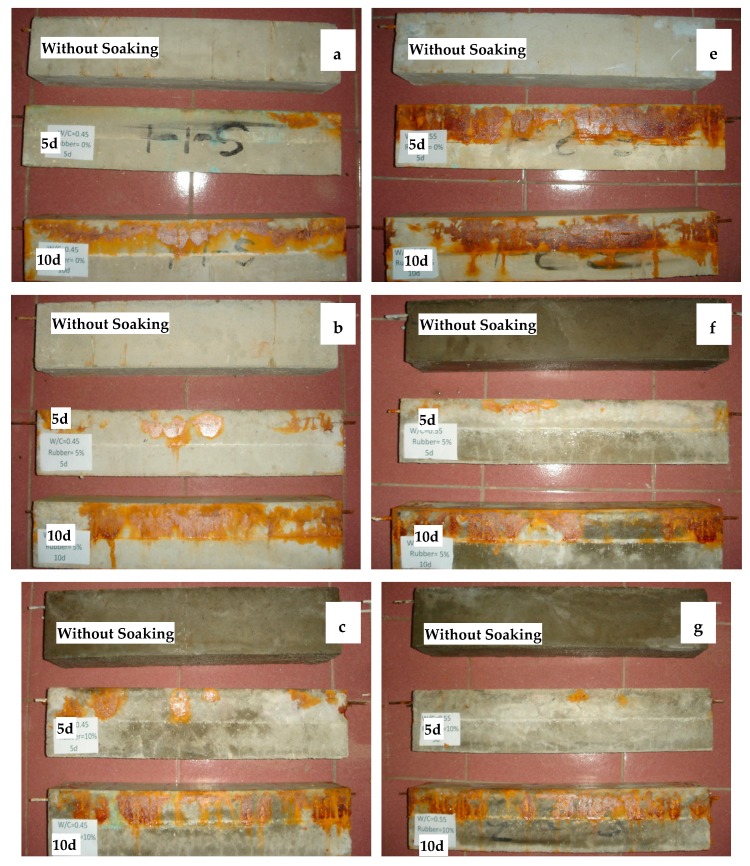

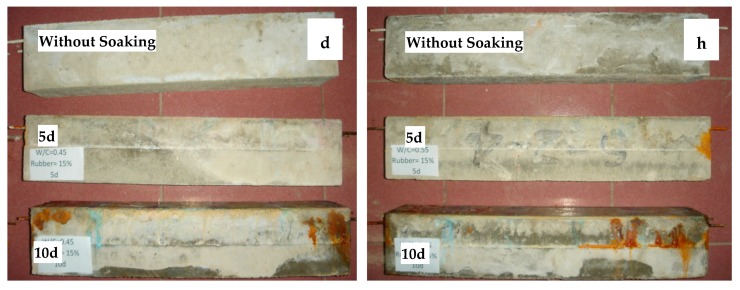
Test specimen after rebar corrosion: (**a**) Reference concrete, W/C = 0.55; (**b**) 50 kg/m^3^ rubber aggregate concrete, W/C = 0.55; (**c**) 100 kg/m^3^ rubber aggregate concrete, W/C = 0.55; (**d**) 150 kg/m^3^ rubber aggregate concrete, W/C = 0.55; (**e**) Reference concrete, W/C = 0.45; (**f**) 50 kg/m^3^ rubber aggregate concrete, W/C = 0.45; (**g**) 100 kg/m^3^ rubber aggregate concrete, W/C = 0.45 and (**h**) 150 kg/m^3^ rubber aggregate concrete, W/C = 0.45.

**Figure 4:**
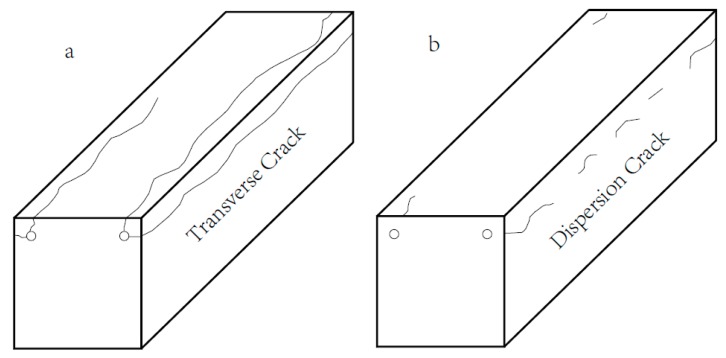
Specimen bursting characteristics: (**a**) Control concrete and (**b**) Rubber aggregate concrete.

The corrosion test specimen is shown in Figure 3. It can be seen that the corrosion area of control concrete specimen surface is greatest. Corrosion area reduced with the increase of rubber aggregate considerably. There is almost no corrosion on the concrete surface while rubber content reaches 150 kg/m^3^. At the same time, it can also be found that longitudinal cracks generated on the side and upper surface of control specimen, as shown in Figure 3a. The crack width value showed that the control concrete in the 5d and 10d corrosion cycles reached moderate and severe corrosion respectively. In contrast, the cracking of rubber aggregate concrete is much less, and the crack width is less than that of the control concrete significantly. In addition, the specimen containing 150 kg/m^3^ rubber did not crack. As shown in Figure 4, for the crack morphology, the rubber aggregate concrete had small, dispersed cracks along the longitudinal direction.

**Figure 5:**
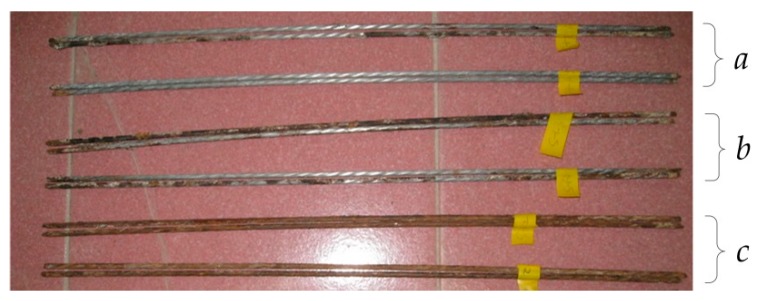
Corrosion of reinforcing steel: (**a**) Slight corrosion; (**b**) Moderate corrosion and (**c**) Severe corrosion.

It is generally considered that spot corrosion occurs under natural environment, and uniform corrosion occurs under the condition of applied electric power. Nevertheless, rebar which was taken out and rusted from the concrete specimen had been slightly corroded on spots, as shown in Figure 5a,b, In the case of severe corrosion, spot corrosion is more serious, as shown in Figure 5c. the rebar cross-section is severely reduced. We believed that the reason for this phenomenon is that: the distribution of pore structure and pore size in the concrete around the rebar is not uniformly, and the saturation of capillary holes is high, this resulted a higher chloride ion concentration than that of surrounding concrete, and then the corrosion increased; Second, the salt solution and oxygen can enter the corrosion area in large quantities and accelerate the local corrosion after concrete cracking.

### 3.2. Potential Changes

**Figure 6:**
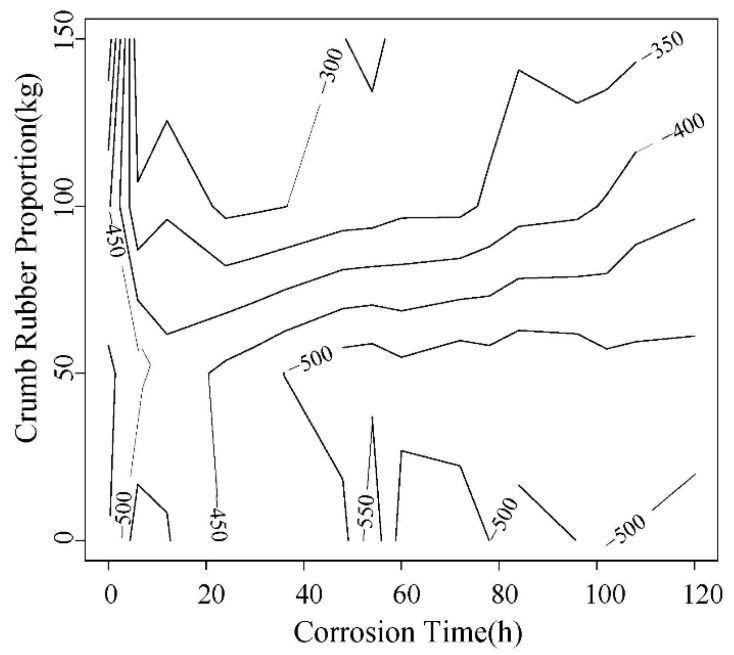
Change trend of the corrosion potential value of steel bar in 0.45 water-cement ratio.

**Figure 7:**
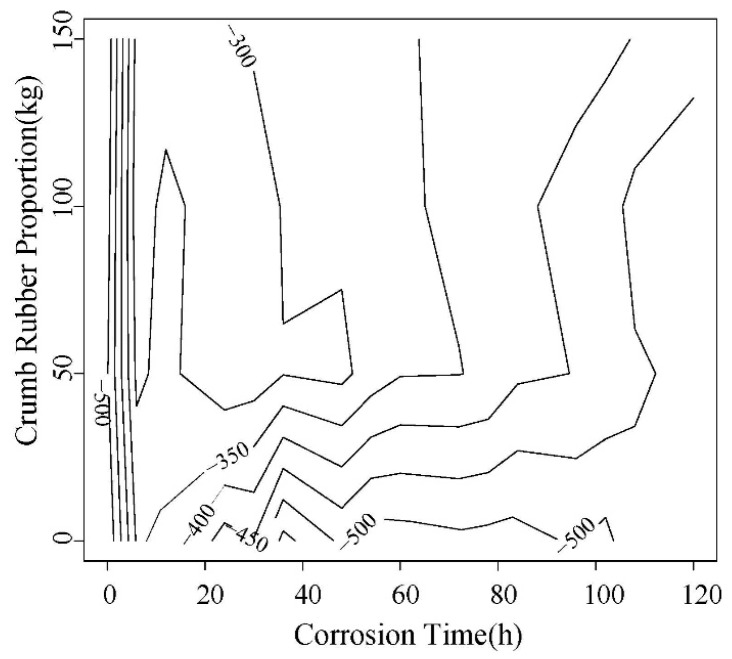
Change trend of corrosion potential of steel bar in 0.55 water-cement ratio.

**Figure 8:**
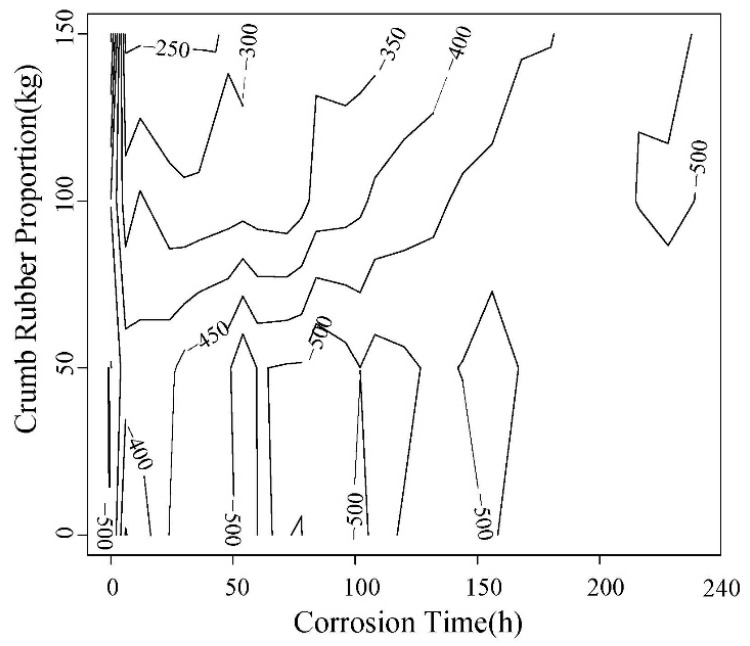
The trend of the corrosion potential value of steel bar in 0.45 water-cement ratio.

**Figure 9:**
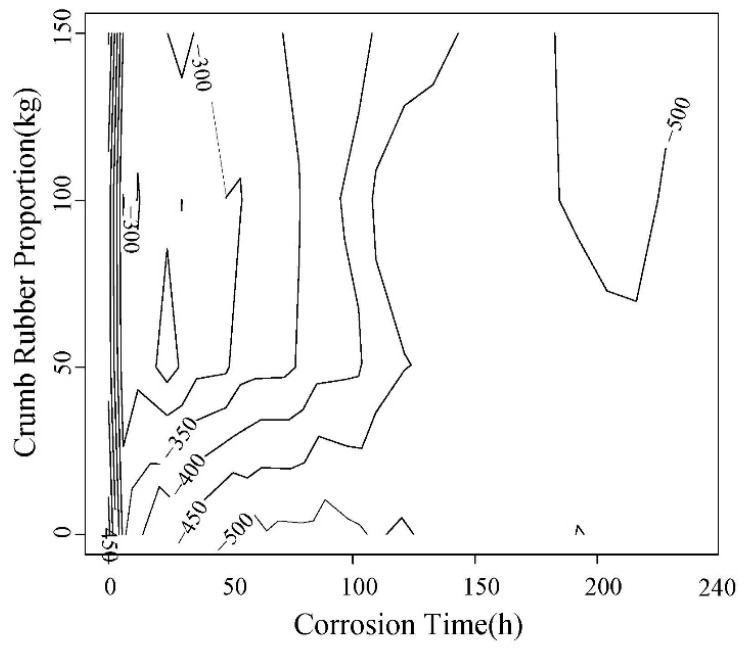
Trend of corrosion potential value of steel bar in 0.55 water-cement ratio.

Rebar corrosion potential values of 5 d power supplying are shown in Figure 6 and Figure 7. The ones of 10 d power supplying are shown in Figure 8 and Figure 9. The greater the distance between two lines in potential diagram, the longer the time required for potential change, the stronger the ability of concrete to resist chloride ion diffusion. As can be seen in Figure 6 and Figure 7, the potential value of the concrete without rubber in 24 h reached −400 MV, and with the increase of rubber content, the potential value is getting smaller in the early stage of the test, and the time required for the potential to reach −500 MV corrosion is longer.

From Figure 6, Figure 7, Figure 8 and Figure 9, after the 5d and 10d power supplying is completed, the potential values of all concrete beams are lower than −350 MV. From the rebar corrosion criteria in this paper, it can be seen that all the concrete beams reach the 95% corrosion state possibly. It can be inferred that all bars have different corrosion. For the same water-cement ratio and power supplying time, the potential value of specimen increased with the rubber content increase, this means that the corrosion degree decreases.

**Figure 10:**
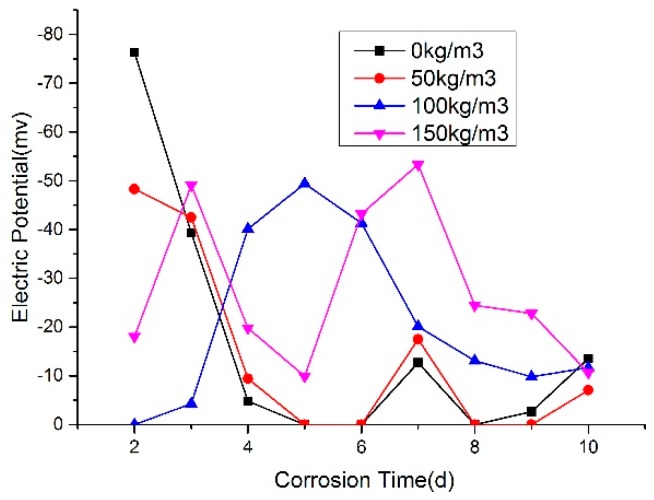
Potential value of W/C = 0.45.

**Figure 11:**
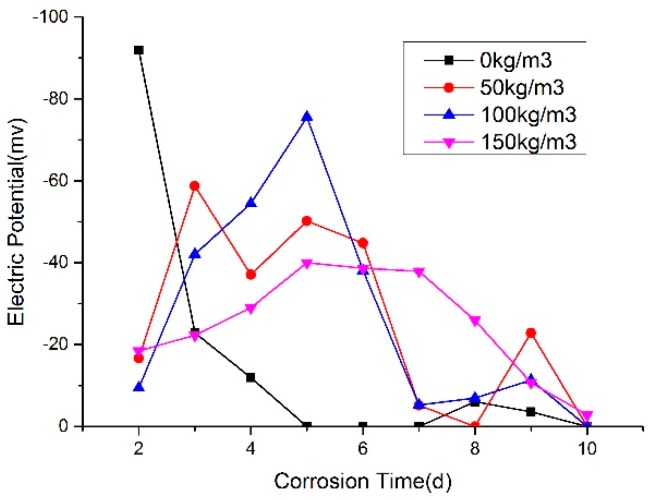
Potential value of W/C = 0.55.

As shown in Figure 10 and Figure 11, it can be found that with the incorporation of rubber, the peak of potential change tends to be delayed, and the maximum change potential shows a decreasing trend.

### 3.3. Mass Loss

After the accelerated corrosion test, the concrete specimens were broken, the bars removed from that were polished using steel brushes and sandpaper to remove residual concrete and rust from the surface. Then they were soaked in dilute sulfuric acid at a concentration of 10% for 15 min, and then they were re-polished until to the fresh surface. Finally, it was placed in an oven to dry, and it was weighed with accuracy of 0.01 g.

**Figure 12:**
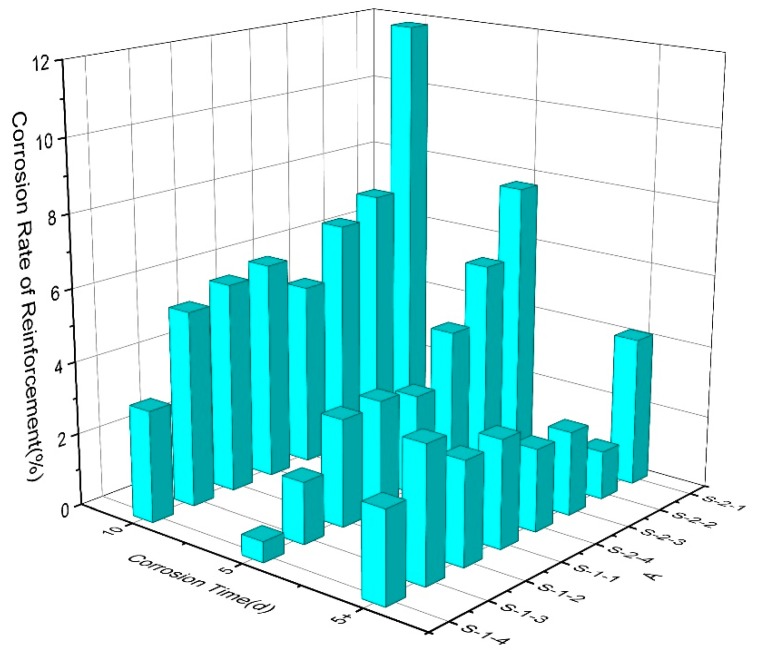
Reinforcement concrete mass loss rate at various levels.

As shown in Figure 12, the mass loss rate of the rebars is the largest when the number of days of corrosion is 10 days. With the increase of the rubber content, the mass loss rate tends to be lower and lower, the same trend for 5 days also. The mass loss rate of rebar with 10 days corrosion and 5 days corrosion was taken as the value of 6–10 days, as shown in column 5 + d. It can be found that the mass loss rate gradually changed from the majority of previous 5 days to the next 5 days with the increase in the amount of rubber, especially S-1-4 and S-1-3 of 0.45 water-cement ratio. This is also agreed with the aforementioned law of segmented change in potential.

As shown in Figure 12, it showed that the mass loss rate of rebar declined with the increase of rubber content under the same water-cement ratio. This is consistent with the potential value trends The mass loss ratio of high water-cement ratio is higher than that of low W/C ratio, which is also consistent with the potential value of rebar.

### 3.4. Results of Flexural Test after Corrosion

After the accelerated corrosion test, four-point bending test was performed as a mechanical index to evaluate effect of chloride ion diffusion on the concrete. Considering the uncertainty of failure load in the test, the load corresponding to 2.5 mm at mid-span and corresponding fracture energy were analyzed in this paper.

(1) Load-Displacement character

**Figure 13:**
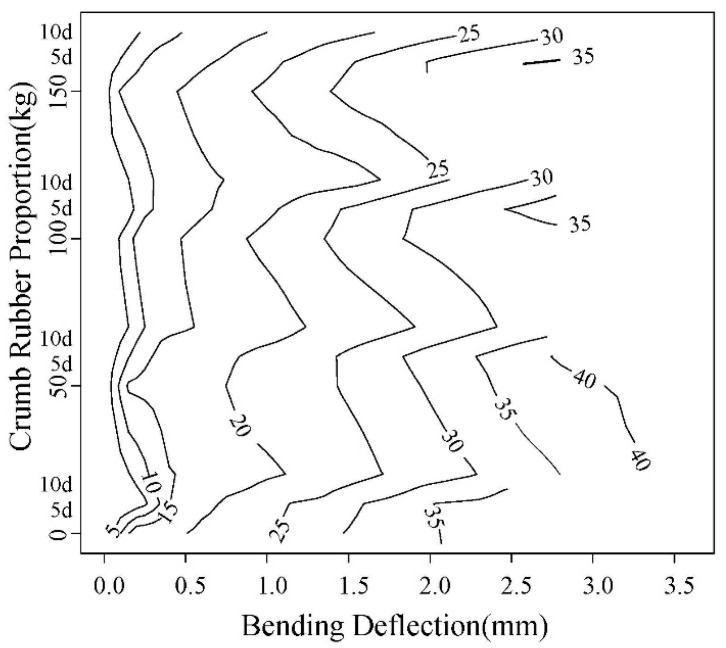
0.45 Water-cement ratio load-displacement contour diagram.

**Figure 14:**
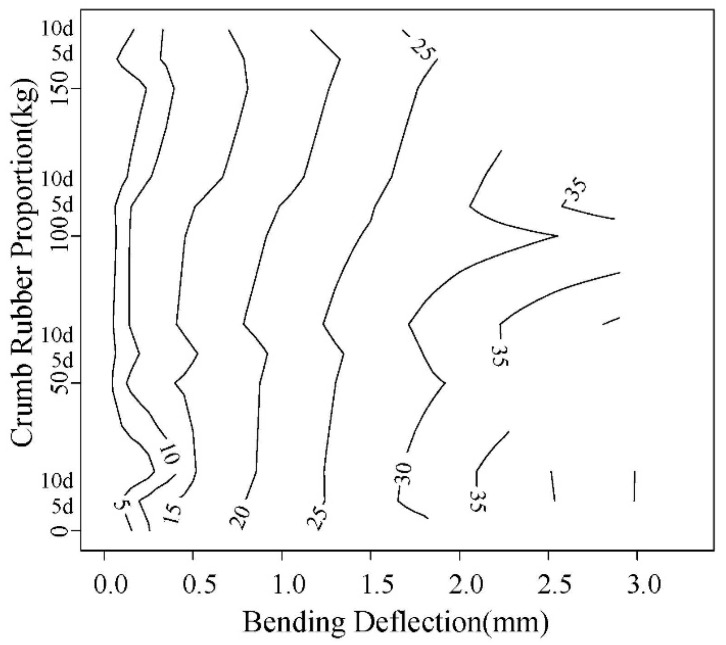
0.55 water-cement ratio load-displacement contour diagram.

The load-displacement contours obtained in the test are shown in Figure 13 and Figure 14. The right line in the figure is the failure displacement. When the spacing between adjacent lines is same, the slope of load-displacement curve is unchanged. It can be seen that the slope before and after 0.3 mm are different. The load-displacement curve shows a linear change at the initial loading stage, and the slope decrease with the corrosion increasing. After the linear segment, the load-displacement abruptly changes, showing that the displacement increases faster than the load. The curve becomes steady, the concrete enters loading stage with cracks, and the point is the initial cracking point. The displacement corresponding to this point is initial cracking displacement.

As can be seen in Figure 13 and Figure 14, with the increase of rubber content, the displacement for same load gradually increases. With the accelerated corrosion, the deformation increases by different degrees.

**Figure 15:**
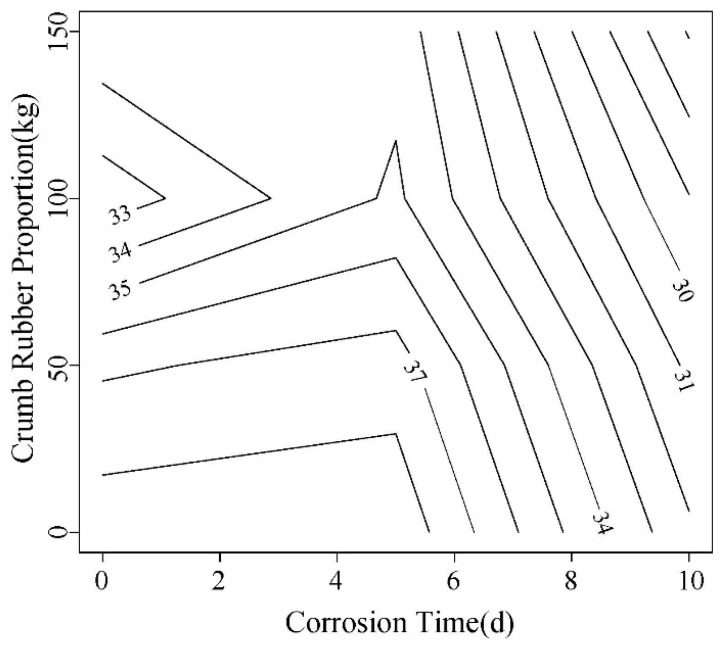
The corresponding load contour maps for 0.45 water-cement ratio 2.5 mm displacement.

**Figure 16:**
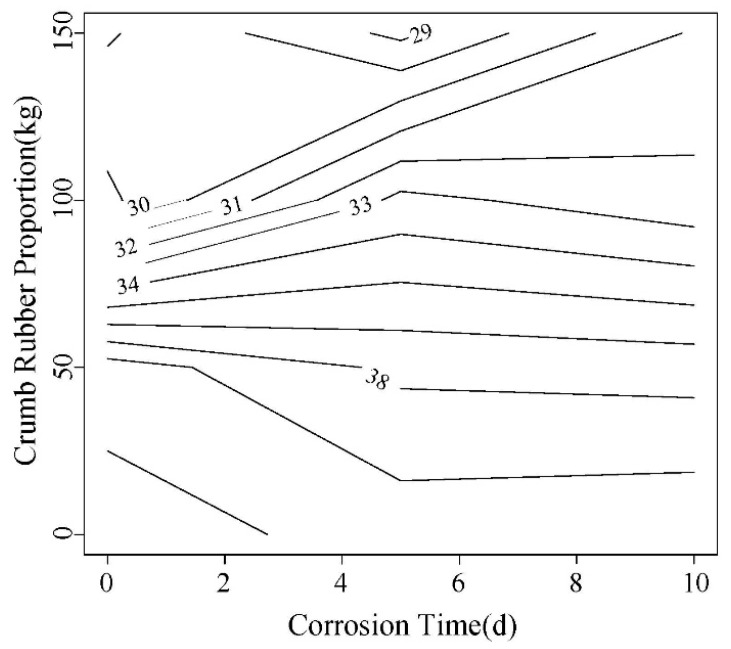
The equivalent load contour map of 0.55 water-cement ratio 2.5 mm displacement.

In this paper, the load corresponding to 2.5 mm is compared. As shown in Figure 15 and Figure 16, this load shows a decreasing trend with the increase of corrosion time. A same trend was observed for rubber content. In addition, the load of high W/C ratio is slightly larger than that of low one.

(2) Unit intensity energy consumption

The incorporation of rubber in the concrete reduces the strength of the concrete, energy dissipation is compared using the ratio of the load corresponding to 2.5 mm to cubic compressive strength. It means the contribution of cubic compressive strength to the anti-cracking property. The larger this parameter is, the greater the contribution is.

**Figure 17:**
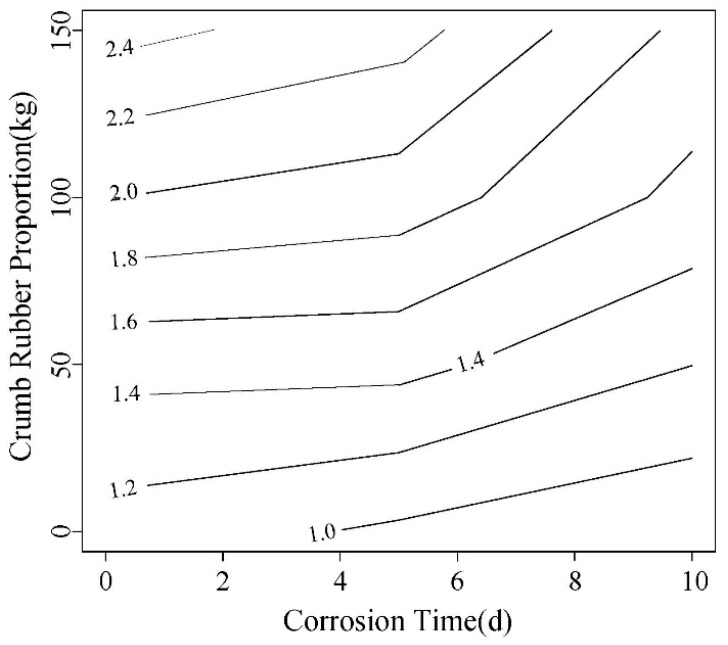
Equipotential line diagram of unit energy consumption corresponding to 2.5 mm displacement at mid-span of 0.45 water-cement ratio.

**Figure 18:**
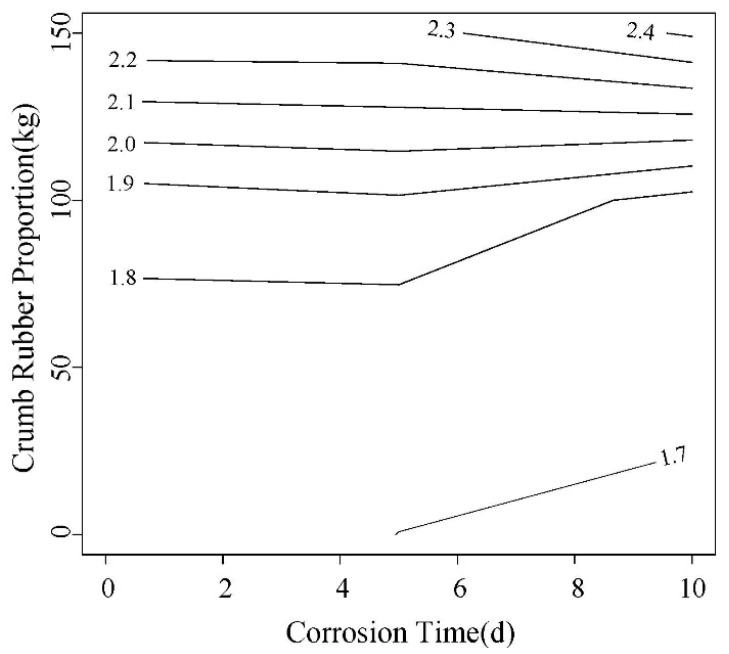
Equipotential line diagram of unit energy consumption corresponding to 2.5 mm displacement at mid-span of 0.55 water-cement ratio.

As shown in Figure 17 and Figure 18, the corresponding unit energy consumption shows an increasing trend as the corrosion time and rubber content increasing. In terms of water-cement ratio, the unit energy consumption of high water-cement ratio is slightly larger than that of low ratio.

## 4. Conclusions

For the concrete facing the chloride salt environment, the rebar corrosion is mainly affected by the capillary pore pressure. Considering the contact angle effect, incorporation of rubber reduces the capillary pores pressure and rebar corrosion. The rebar corrosion was delayed and reduced.

The cracks of rubber concrete are dispersed along the longitudinal direction, it is different with the completely crack of control concrete.

Rebar corrosions of rubber aggregate concrete and control concrete are spot corrosion. Concrete with a higher water-cement ratio will have a better anti-corrosion performance.

The load-displacement curve shows that the bearing capacity loss of the concrete beam basically decreases with the increase of rubber aggregate. With the increase of the corrosion cycle, the loss of bearing capacity increases, and the amount of 150 kg/m^3^ of rubber aggregate concrete is less than 5%.

Adding rubber aggregate into concrete opens a new approach to address the rebar corrosion problem.

## Figures and Tables

**Table 1:** Ratio of rubber aggregate concrete beams.

No.	W/C Ratio	Cement Content (kg/m^3^)	Water Content (kg/m^3^)	Sand Rate (%)	Sand Content (kg/m^3^)	Aggregate (kg/m^3^)	Rubber Particles (kg/m^3^)	Water Reducer (kg/m^3^)
S-1-1	0.45	400	180	0.40	718	1076	0	2
S-1-2				0.37	588	1076	50	2
S-1-3				0.34	458	1076	100	2
S-1-4				0.31	328	1076	150	2
S-2-1	0.55	400	220	0.40	676	1014	0	2
S-2-2				0.37	546	1014	50	2
S-2-3				0.34	416	1014	100	2
S-2-4				0.30	286	1014	150	2
